# MaterniCode: New Bioinformatic Pipeline to Detect Fetal Aneuploidies and Rearrangements Using Next-Generation Sequencing

**DOI:** 10.1155/2024/8859058

**Published:** 2024-06-13

**Authors:** Federico Gabrielli, Filomena Tiziana Papa, Fabio Di Pietro, Andreu Paytuví-Gallart, Daniel Julian, Walter Sanseverino, Cinzia Alfonsi

**Affiliations:** ^1^ Biolab srl, Laboratorio di Genetica molecolare e Genomica 63100, Ascoli Piceno, Italy; ^2^ Sequentia Biotech SL, C/del Dr. Trueta, 179 08005, Barcelona, Spain

## Abstract

**Objective:** The present study is aimed at introducing and evaluating MaterniCode, a state-of-the-art bioinformatic pipeline for noninvasive prenatal testing (NIPT) that leverages the Ion Torrent semiconductor sequencing platform. The initiative strives to revolutionize prenatal diagnostics by offering a rapid and cost-effective method without sacrificing accuracy.

**Methods:** Two distinct bioinformatic strategies were employed for fetal sex determination, one of which achieved 100% accuracy. We analyzed 1225 maternal blood samples for fetal aneuploidies, benchmarking against the industry standard Illumina VeriSeq™ NIPT Solution v2. The capability of MaterniCode to detect and characterize complex chromosomal anomalies was also assessed.

**Results:** MaterniCode achieved near-perfect accuracy in fetal sex determination through chromosome Y (chrY )–specific gene analysis, whereas the alternative method, employing the ratio of high-quality mapped reads on chrY relative to all reads, delivered 100% accuracy. For fetal aneuploidy detection, both the integrated WisecondorX and NIPTeR algorithms demonstrated a 100% sensitivity and specificity rate, consistent with Illumina VeriSeq™ NIPT Solution v2. The pipeline also successfully identified and precisely mapped significant chromosomal abnormalities, exemplified by a 2.4 Mb deletion on chromosome 13 and a 3 Mb duplication on chromosome 2.

**Conclusion:** MaterniCode has proven to be an innovative and highly efficient tool in the domain of NIPT, demonstrating excellent sensitivity and specificity. Its robust capability to effectively detect a wide range of complex chromosomal aberrations, including rare and subtle variations, positions it as a promising and valuable addition to prenatal diagnostic technologies. This enhancement to diagnostic precision significantly aids clinicians in making informed decisions during pregnancy management.

## 1. Introduction

The realm of prenatal screening and diagnostics has undergone transformative progress with the advent of noninvasive prenatal testing (NIPT). This innovative technology harnesses cell-free fetal DNA (cffDNA) from maternal plasma to primarily detect fetal aneuploidies of chromosomes 13, 18, and 21. Seminal research from 2008 has underscored the promise of NIPT, with subsequent studies expanding its scope to encompass additional autosomal aneuploidies and segmental chromosomal aberrations [1–6].

The technological spectrum within NIPT is broad: some methodologies employ shallow whole-genome sequencing to procure a comprehensive genomic snapshot, whereas others target specific loci—predominantly chromosomes 13, 18, and 21—through targeted enrichment strategies [1–9]. Notably, although Illumina's sequence-by-synthesis technology has been predominant, recent advancements have validated the efficacy of semiconductor sequencing in fetal aneuploidy detection [10].

A pivotal aspect of NIPT's accuracy is the fetal DNA fraction present in the sample, which is influenced by variables such as gestational age and maternal weight. Ensuring a sufficient fetal fraction is vital for the test's sensitivity. Conventionally, the presence of the Y chromosome (FF-Y) in plasma samples is indicative of male pregnancies, yet this method is limited to such cases. Alternative strategies, which differentiate between maternal and fetal DNA based on single nucleotide polymorphisms, often entail additional costs due to supplementary testing [11, 12].

The introduction of seqFF by Kim et al., which calculates the fetal fraction from existing NIPT data, stands out for its efficiency, eliminating the need for extra tests and extending its utility to pregnancies of either sex [13]. Despite such strides, discrepancies between NIPT results and standard fetal karyotypes present ongoing challenges, with factors such as placental mosaicism and maternal copy number variations contributing to these inconsistencies [14–16].

Emphasizing the importance of this field, fetal congenital anomalies continue to be a leading cause of perinatal morbidity, with approximately 1 in 150 live births exhibiting chromosomal abnormalities [17]. While current invasive diagnostic methods are effective, they carry a risk of miscarriage [18]. As a less risky alternative, the exploration and analytical potential of cffDNA in maternal plasma have heralded a new epoch in prenatal screening, diminishing reliance on invasive techniques [19–21].

The emergence of NIPT offers a safer route for fetal aneuploidy detection through the analysis of cffDNA in maternal blood. Crucial to this analysis is the proportion of fetal DNA within the total cell-free DNA pool, a metric subject to various maternal and fetoplacental factors. Suboptimal fetal DNA concentrations, particularly below the 4% threshold, pose significant challenges during quality control, increasing the risk of false negatives. In the wake of next-generation sequencing and advanced bioinformatics, multiple strategies for predicting fetal aneuploidies have come to the fore. This paper presents MaterniCode, an integrated pipeline that includes fetal fraction estimation, gender prediction, chromosomal aneuploidy prediction, and CNV analysis, leveraging the semiconductor sequencing capabilities of the Ion Torrent platform.

## 2. Materials and Methods

### 2.1. Patient Cohort

The study included 1225 expectant mothers, each beyond the 10-week gestation mark, carrying a single fetus. Following postinvasive testing, all participants either had normal aneuploid karyotypes or received confirmation of normal results from a secondary NIPT via the Illumina VeriSeq™ NIPT Solution v2. Informed consents were obtained after a comprehensive briefing about the study. For each participant, 10 mL of blood was collected in Cell-Free DNA BCT™ tubes prior to any invasive procedures.

### 2.2. Sample Collection, DNA Isolation, Sequencing, Quality Control, and Aneuploidy Prediction

A control dataset comprising 140 samples, all confirmed to have normal prenatal karyotypes, was established. This dataset served as a benchmark for the fetal aneuploidy analysis using WisecondorX and NIPTeR tools.

Collected blood samples were processed within 24 h. The process involved centrifugation at 1600 × g for 20 min at 4°C to separate plasma. The supernatant was then stored at −20°C. The cffDNA extraction from 2 mL of plasma utilized a silica-coated magnetic bead-based method.

Library preparation followed the Ion Plus Fragment Library Kit (Thermo Fisher) protocol with minor modifications. The sequencing was executed on the Ion S5 system, employing 500 flow cycles.

Raw reads from the Ion Torrent sequencing were first assessed. Quality checks were conducted using FastQC, and sequences were trimmed using Trimmomatic (version 0.39) to exclude those below 35 bp or with a *Q* score under 20. Duplicate sequences were removed to ensure data integrity.

The high-quality, filtered sequences were aligned against the complete human genome (GRCh37) using the Burrows–Wheeler Aligner (BWA-mem) [22].

We employed both WisecondorX and NIPTeR in our post-NIPT analysis to predict aneuploidies. This dual approach was specifically designed to mitigate biases and enhance the accuracy of the results [23, 24].

To estimate fetal fraction, the DEFRAG3 and SeqFF algorithms were applied. DEFRAG3 was primarily used for male fetuses, focusing on Y chromosome sequences. SeqFF was employed for both genders, utilizing elastic net (Enet) and weighted rank selection criterion (WRSC) models [13, 25].

## 3. Results

### 3.1. Fetal Sex Determinations

In this study, we employed two distinct methods for fetal sex determination:

#### 3.1.1. General Chromosome Y (chrY) Read Analysis

**Figure 1:**
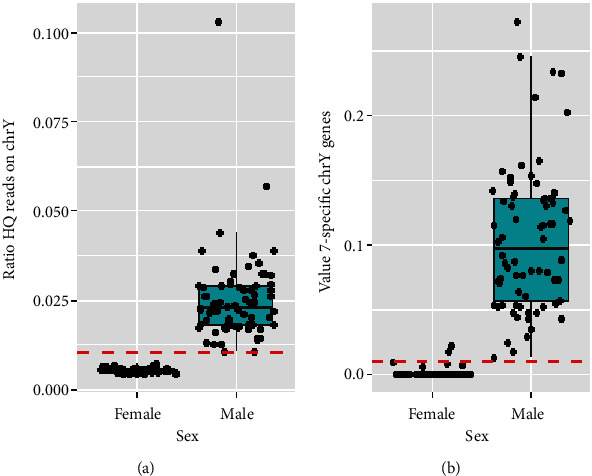
(a) The ratio of high-quality mapped reads between male and female samples meeting our quality criteria (4% FF, 1.5 M of reads). A significant cluster of female samples approaches a value of 0, with most positioned below the 0.01 threshold, emphasizing the method's accuracy. (b) The analysis of the seven specific genes on chromosome Y. Based on this analysis, the majority of samples accurately determined sex, with a small percentage inaccurately predicted. This highlights the precision and effectiveness of our pipeline's methodologies in fetal sex determination.

We also analyzed the ratio of high-quality mapped reads on chrY relative to all reads. This approach's results are visualized in Figure 1(a), displaying the distribution of filtered high-quality mapped reads for fetal sex determination. Here, a clear demarcation is observed with most female samples clustering near a value of 0, indicated by a horizontal red dotted line at a threshold score of 0.01. The majority of females fell below this threshold, highlighting the method's high accuracy (approximately 100%) in determining fetal sex.

#### 3.1.2. Analysis Based on chrY Genes

We evaluated the ratio of reads that mapped onto seven specific genes on chrY compared to all reads mapping to chrY. The selection of seven genes for fetal sex determination likely reflects their Y-chromosome specificity, functional importance, and diagnostic reliability [26]. This method's efficacy is graphically represented in Figure 1(b), which illustrates the values derived for these seven genes. Approximately 98% of the samples accurately determined fetal sex, with a small fraction (~2%) yielding incorrect predictions.

Adjusting the threshold values could enhance accuracy, albeit with trade-offs in male and female determinations.

Postevaluation, the method focusing on mapping high-quality reads on the Y chromosome demonstrated near-perfect accuracy. This methodology, alongside the chrY gene-based analysis, has been integrated into our pipeline, ensuring both heightened sensitivity and specificity for fetal sex determinations. Despite the trade-offs in adjusting threshold values, both methods exhibit exceptional performance, with the chrY read analysis standing out for its remarkable accuracy.

### 3.2. Sex Chromosome Aneuploidy (SCA) Examination

#### 3.2.1. For Females

**Figure 2:**
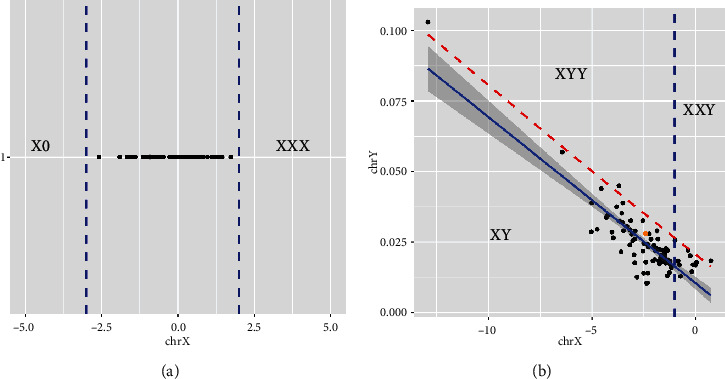
Composite one-dimensional graph displaying chromosomal analysis for sex determination; (a) *X*-axis represents *Z*-score for mapped X chromosome reads in females against healthy references. This helps in identifying any deviations indicative of aneuploidies. (b) For male samples, a scatter plot is shown where the *X*-axis indicates the percentage of reads aligned on the X chromosome and the *Y*-axis captures the percentage of reads mapped on the Y chromosome, specifically from the seven distinctive regions of chromosome Y, thereby excluding the PAR. This dual-axis representation assists in clearly differentiating male samples based on their unique chromosomal mapping patterns.

The examination involved comparing the percentage of reads mapped on the X chromosome of the test sample against those in healthy reference samples. This comparison allowed for the identification of potential aneuploidies specific to the X chromosome in female fetuses (Figure 2(a)).

#### 3.2.2. For Males

The analysis was more multifaceted. It included a comparison on two axes: the *X* axis represented the percentage of reads aligned on the X chromosome, while the *Y* axis depicted the percentage of reads mapped on the Y chromosome, excluding shared areas like the pseudoautosomal regions (PAR) (Figure 2(b)). The calculations for the *Y*-axis were specifically derived from mapping onto the seven specific regions of chrY. Through these tailored analytical approaches for both sexes, the study efficiently addresses the complexities of detecting SCAs. The data visualization in Figure 2 plays a pivotal role in elucidating these differences and enhancing the accuracy of SCA detection.

### 3.3. Fetal Aneuploidy Detection

In our study, we analyzed a total of 1225 samples, among which some displayed fetal trisomy, sex chromosome alterations, and CNVs (Table 1). Prior examination of these samples was done using the Illumina VeriSeq™ NIPT Solution v2. Our pipeline, integrating both WisecondorX and NIPTeR algorithms, accurately identified these chromosomal anomalies, achieving a sensitivity and specificity rate of 100%. All aneuploidies were confirmed by NIPTeR as well. Fetal fractions were also compared with Illumina analysis, showing complete concordance with a deviation of ± 0.8%.

### 3.4. WisecondorX Analysis

**Figure 3:**
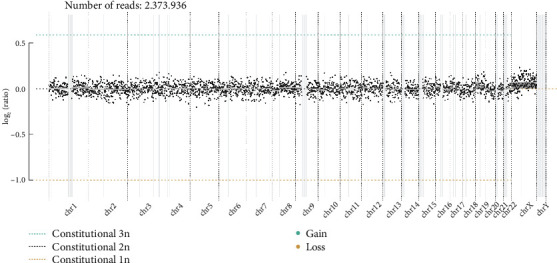
Normal fetal karyotype predicted by WisecondorX. Plot provides a detailed visualization of chromosomal aneuploidies by plotting data in 1-Mb bins. Each dot's vertical position on the graph represents the log2-transformed ratio of observed to expected reads, indicating chromosomal stability or anomalies. Dot size reflects the certainty of each observation, while segments with varying line widths represent areas of predicted equal copy number, aiding in the detection and analysis of specific chromosomal variations.

WisecondorX provided detailed insights into chromosomal aneuploidies, segmenting data into bins of 1 Mb. This level of granularity is depicted in Figure 3, offering a clear and detailed visualization of chromosomal variations. The graph illustrates the normal fetal karyotype predicted by WisecondorX and aids in identifying specific chromosomal anomalies.

### 3.5. Comparative Analysis and Validation

As shown in Figure 3, our pipeline's analysis of the patient cohort identified multiple cases of aneuploidy. Each of these findings was corroborated by the Illumina VeriSeq™ NIPT Solution v2, indicating a consistent overlap and affirming the reliability and accuracy of our analytical approach.

**Figure 4:**
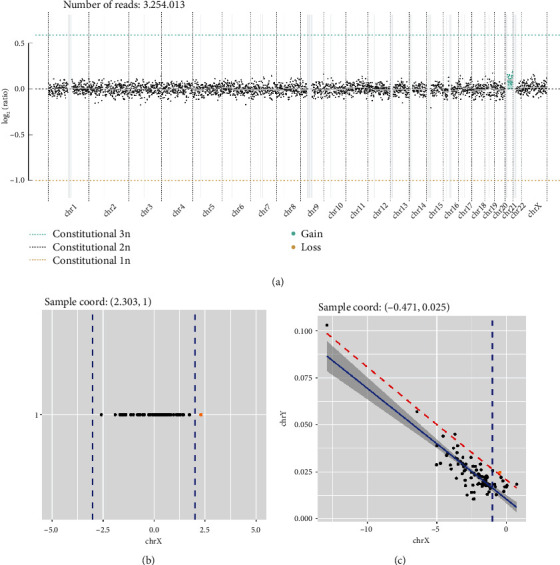
Analysis of aneuploidy detection using our pipeline: (a) WisecondorX method detailing read counts across various chromosomes of T21 sample; (b) visualization of detected XXX aneuploidy case from our patient cohort; (c) visualization of detected XXY aneuploidy case from our patient cohort. The consistent overlap in findings showcases the reliability and accuracy of our analytical approach.

Figure 4(a) presents the WisecondorX method detailing read counts across various chromosomes of a T21 sample.

Figure 4(b) visualizes a detected XXX aneuploidy case from our cohort.

Figure 4(c) illustrates a detected XYY aneuploidy case from our cohort.

In one intriguing case, the VeriSeq™ NIPT Solution v2 identified an aneuploidy on chromosome 13, whereas our pipeline indicated a normal chromosome. Subsequent conventional diagnostic (fetal karyotype) methods validated the accuracy of our pipeline's prediction.

### 3.6. Identification of Chromosomal Abnormalities

Our pipeline also successfully detected intricate chromosomal abnormalities, such as a 2.4 Mb deletion on chromosome 13 and a 3 Mb duplication on chromosome 2. These findings were confirmed through additional diagnostic methods such as Array-CGH.

**Figure 5:**
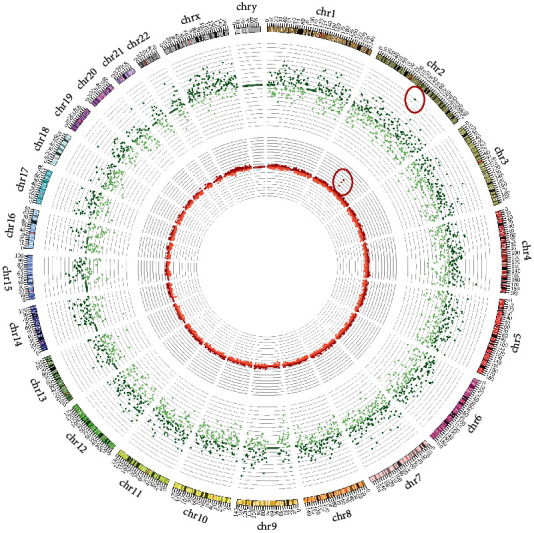
Circos plot displaying *z*-scores in the inner part and log ratios in the outer ring for each chromosome in a sample with a 3 Mb duplication.

**Figure 6:**
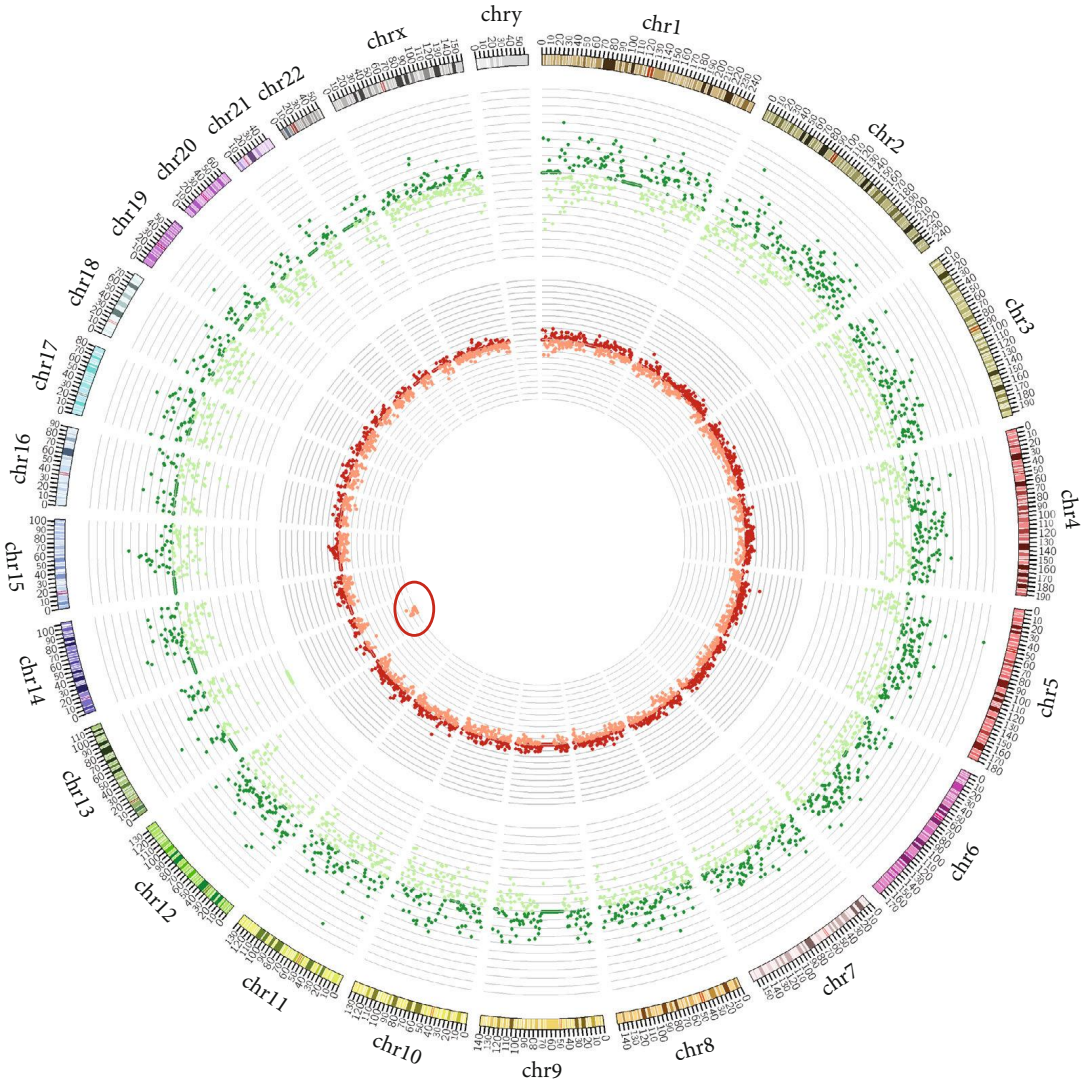
Circos plot displaying *z*-scores in the inner part and log ratios in the outer ring for each chromosome in a sample with a deletion on chromosome 13.

For an enhanced visual representation, the read distribution and *z*-scores for each sample were plotted in circos diagrams, as shown in Figures 5 and 6. These plots provide a comprehensive view of the read distributions and *z*-scores, facilitating the discernment of chromosomal duplications and deletions.

Figure 5 is a circos plot illustrating the *z*-score and read distribution for each chromosome in a sample with a 3 Mb duplication.

Figure 6 presents a similar circos plot for a sample with a deletion on chromosome 13.

Through these analyses, our pipeline not only confirmed the results obtained through conventional methods but also demonstrated its robust capability to detect and accurately characterize both common and complex chromosomal anomalies in prenatal samples.

## 4. Discussion

The evolution of NIPT has been a game-changer in the field of prenatal diagnostics. Our study is aimed at refining this process further through the introduction of a novel pipeline, MaterniCode. The results obtained from our research highlight the exceptional accuracy of this new approach.

One of the most notable aspects of MaterniCode is its remarkable sensitivity and specificity, both achieving a 100% rate. This level of precision in identifying true positives and ruling out negatives is of critical importance in prenatal diagnostics, where the stakes of misdiagnosis are exceptionally high.

When compared with the established Illumina VeriSeq™ NIPT Solution v2, our pipeline's results were largely consistent with Illumina's findings. A notable exception was an instance involving chromosome 13 aneuploidy, where our pipeline differed from Illumina's analysis but was later validated by conventional diagnostic methods. This case underscores the need for diverse and independent analysis methods in prenatal diagnostics, highlighting potential limitations in relying solely on existing methodologies.

The ability of our pipeline to detect complex chromosomal abnormalities, such as a 2.4 Mb deletion on chromosome 13 and a 3 Mb duplication on chromosome 2, is particularly commendable. Historically, NIPT has been used to identify larger chromosomal anomalies, but our pipeline demonstrates the capability to accurately detect smaller chromosomal aberrations.

The use of circos diagrams in presenting our results provided an effective visual representation of the chromosomal landscape, facilitating intuitive data interpretation. Such visualization techniques are not only beneficial for clinicians in understanding complex genetic data but also serve as a valuable tool in communicating these results to expecting parents, an integral part of prenatal care.

Moreover, MaterniCode's streamlined analysis process allows for fewer sample requirements, reducing both costs and turnaround times. This efficiency opens up the possibility for many more laboratories to adopt this technology, significantly expanding access to high-quality prenatal testing.

In sum, MaterniCode positions itself as a formidable addition to the NIPT toolkit. Although our findings are encouraging, further research involving larger and more diverse cohorts is necessary to validate its universal applicability. As prenatal diagnostics continues to advance, innovative approaches like MaterniCode will be vital in delivering precise, timely, and comprehensive genetic information to expecting parents.

## 5. Conclusions

In the rapidly advancing field of NIPT, our study introduces MaterniCode, a pipeline noted for its high accuracy and reliability. With both sensitivity and specificity rates at 100%, the pipeline represents a notable advancement in prenatal diagnostics.

MaterniCode's ability to detect intricate chromosomal aberrations, such as minute deletions and duplications, demonstrates its advanced diagnostic capability, going beyond the conventional scope of NIPT. The concordance of our pipeline's results with those of established platforms, particularly Illumina VeriSeq™ NIPT Solution v2, is a testament to its robustness and validity in clinical settings.

The utilization of circos diagrams for data visualization has enhanced the interpretability of complex genetic information. This advancement is not only a boon for clinicians in their diagnostic processes but also aids in effectively communicating results to expecting parents, thereby improving the overall experience of prenatal care. While the current findings showcase the potential of our pipeline, MaterniCode is mature enough for clinical use. We are optimistic that MaterniCode and similar innovations will lead the way in realizing safer, more accurate, and comprehensive prenatal testing methodologies. As the landscape of prenatal diagnostics continues to evolve, such advancements will play a crucial role in enhancing the standard of prenatal care, offering expecting parents more reliable and informative genetic insights into their unborn child's health.

## Figures and Tables

**Table 1:** Diagnostic performance of our integrated pipeline.

**Condition**	**Cases**	**TP**	**FP**	**FN**	**PPV%**	**NPV%**
Duplication > 1 mb	8	7	1	0	87.5	100
Deletion > 1 mb	3	3	0	0	100	100
T21	9	9	0	0	100	100
T22	1	1	0	0	100	100
T15	1	1	0	0	100	100
T9	1	1	0	0	100	100
T13	1	1	0	0	100	100
T7	1	1	0	0	100	100
XXX	1	1	0	0	100	100
XYY	1	1	0	0	100	100

*Note:* This table summarizes the performance of our diagnostic pipeline using WisecondorX and NIPTeR algorithms for detecting various chromosomal anomalies in 1225 samples. The table provides a breakdown of cases for each condition along with true positive (TP), false positive (FP), false negative (FN), positive predictive value (PPV%), and negative predictive value (NPV%).

## Data Availability

The data that support the findings of this study are available on request from the corresponding author. The data are not publicly available due to privacy or ethical restrictions.
